# Advances in targeting the folate receptor in the treatment/imaging of cancers

**DOI:** 10.1039/c7sc04004k

**Published:** 2017-12-18

**Authors:** Marcos Fernández, Faiza Javaid, Vijay Chudasama

**Affiliations:** a Department of Chemistry , University College London , London , UK; b Research Institute for Medicines (iMed.ULisboa) , Faculty of Pharmacy , Universidade de Lisboa , Lisbon , Portugal . Email: v.chudasama@ucl.ac.uk

## Abstract

The folate receptor (FR) is a recognised biomarker for tumour cells due to its overexpression on a large number of tumours. Consequently, the FR has been exploited by many diagnostic and therapeutic tools to allow targeted delivery to, and imaging of, cancer cells. Herein, we describe the many different approaches by which this has been achieved, including the attachment of folate to potent chemotherapeutic drugs to form FR-targeting small molecule–drug conjugates (SMDCs), FR-targeting antibodies (as antibody alone and as an antibody–drug conjugate), and in the form of complementary nanotechnology–folate platforms; as well as imaging variants thereof. The potential of exploiting the FR for targeted therapy/imaging has the potential to revolutionise the way several cancers are treated. These FR-targeted technologies can also pave the way for inspiring further sophisticated drug conjugates, especially as this receptor is being targeted by use of several complementary technologies: small molecule, nanoparticle and protein-based – thus providing broad and distinct knowledge in the area.

## Abstract 2

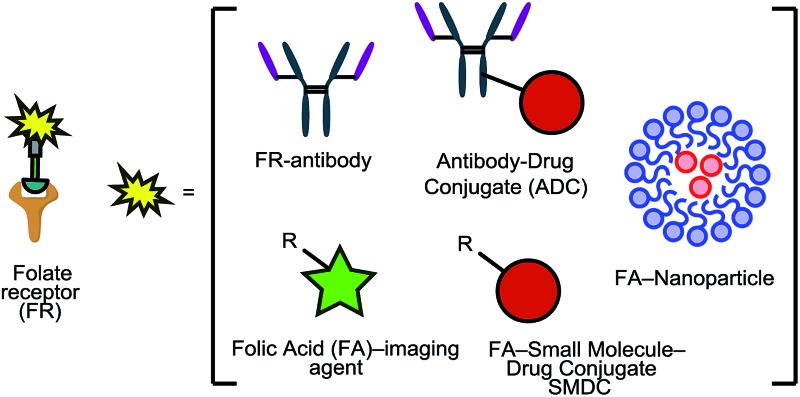
The folate receptor (FR) is an important biomarker for many cancers, and its overexpression on tumours can be exploited for targeted therapy, diagnosis and imaging.

## 1 Introduction

### 1.1 Cancer treatment: chemotherapy and targeted therapy

Tumour-targeted drug delivery systems (TTDDSs) have emerged as a promising strategy in cancer treatment as they largely bypass the adverse side effects characteristic of conventional chemotherapy.^1^ Targeting particular biomarkers that are overexpressed specifically on tumour cells enables the selective delivery of cytotoxic cargo to cancerous tissue, thereby minimising toxic side effects in the patient and increasing the therapeutic index.^2^,^3^ In recent years, many receptors have been identified as being overexpressed on cancer cells, *e.g.* prostate-specific membrane antigen (PSMA), and the carbonic anhydrase IX and biotin receptors.^2^,^4^,^5^ In addition to these, the folate receptor (FR) has attracted considerable attention in the field. This review will highlight the recent progress in the use of the FR, particularly FRα as this is what the vast majority of FR-targeted drug delivery systems have focused on, as a promising candidate for tumour-targeted delivery due to its elevated expression on various cancer cell types.

### 1.2 The folate receptor

Several criteria must be considered when selecting an appropriate receptor for targeted drug delivery, the most important being considerably higher expression of the receptor of interest on cancerous tissue relative to normal cells. Additionally, in receptor-mediated internalisation pathways, the receptor would ideally rapidly recycle back to the cell surface, and/or be upregulated, post interaction with the TTDDS to enable maximum delivery of further targeted therapeutics.^6^ Furthermore, to ensure specific delivery to the target cell, the receptor must be preferentially enriched at its surface and not released in circulation.^7^

Amongst the receptors that meet these requirements is a family of glycoproteins (35–40 kDa) known as the folate receptors (FRs),^8^ which can be divided into three different isoforms: FRα, FRβ and FRγ. The α and β variants are attached to the cell membrane *via* glycosylphosphatidylinositol (GPI) anchors, whereas FRγ is found only in hematopoietic cells^9^ and lacks the GPI component, making it freely soluble.^8^,^10^

The FRβ isoform shares approximately 70% of its homology with FRα, and both possess a comparable affinity for folate.^11^ FRβ is upregulated on activated myeloid cells (primarily monocytes and macrophages) that participate in inflammatory and autoimmune diseases.^12^–^15^ The FRβ isoform has also been detected in tumour-associated macrophages (TAMs) of many cancers, including those of the liver, kidney, skin, lung, blood and soft tissue.^16^–^18^ These macrophages can penetrate solid tumours and promote their metastasis and growth by suppression of CD8^+^ T cells and secretion of proangiogenic factors.^19^ The FRβ isoform can consequently serve as a potential target for the selective delivery of cytotoxic agents in cancer treatment.

**Fig. 1:**
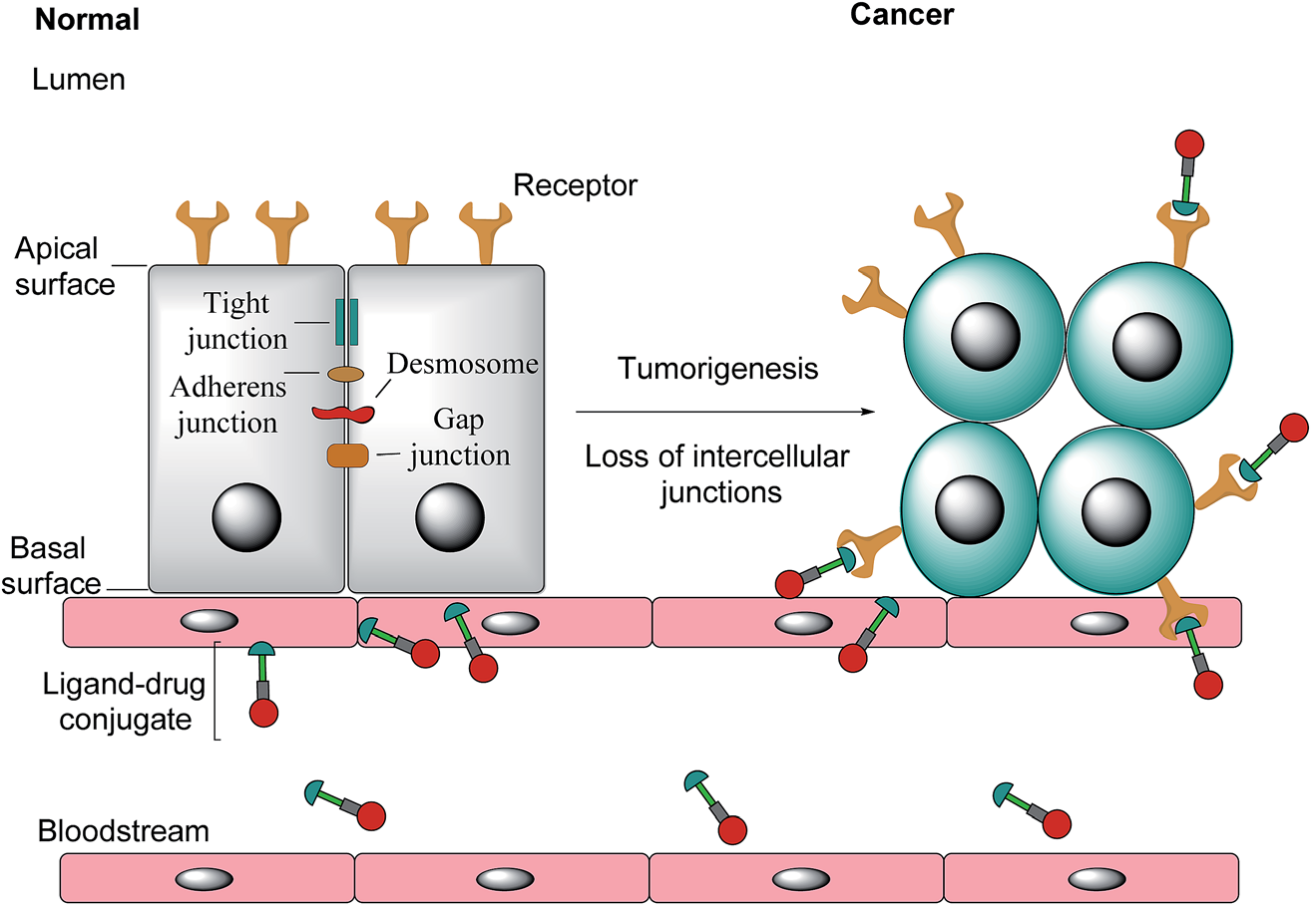
The transformation of healthy epithelial cells to tumour cells and the effect it has on receptor positioning. Upon tumorigenesis, intercellular junctions (tight junctions, adherens junction, desmosomes and gap junctions) are lost and receptors that were previously only found on the apical surface become randomly positioned on the tumour.^6^

Notwithstanding FRβ's expression on some cancers, the FRα isoform has the most potential for targeted cancer therapy as it is the most widely expressed of all the FR isoforms^20^ and is overexpressed in a large number of cancers of epithelial origin, including breast,^21^ lung, kidney and ovarian cancers,^22^ the expression in these carcinomas being 100–300 times higher than on healthy cells and in the order of 1–10 million receptor copies per cell.^23^,^24^ Most healthy cells use the reduced folate carrier for folate uptake and thus the expression and distribution of FRα in non-cancerous tissue is largely confined to cells crucial for embryonic development, the choroid plexus in the brain and to the kidneys, where folates are filtered through the glomeruli and subsequently reabsorbed in the proximal tubule cells *via* FR-binding.^8^,^25^,^26^ Moreover, only the apical surface of these healthy cells expresses FRα, prohibiting the exposure of this receptor to folates found in circulation and cytotoxic agents administered parenterally, as intercellular junctions impede small molecules from crossing the epithelium.^6^,^27^ Upon tumorigenesis however, the entire architecture of a cell changes, with the vasculature becoming disorganised and chaotic and intercellular junctions being lost, giving rise to weak association between endothelial cells.^28^ As a consequence, FRα loses its polarised cellular localisation and becomes randomly distributed over the entire cell surface.^6^ This phenomenon now renders FRα accessible to drug conjugates in blood circulation (Fig. 1).

**Fig. 2:**
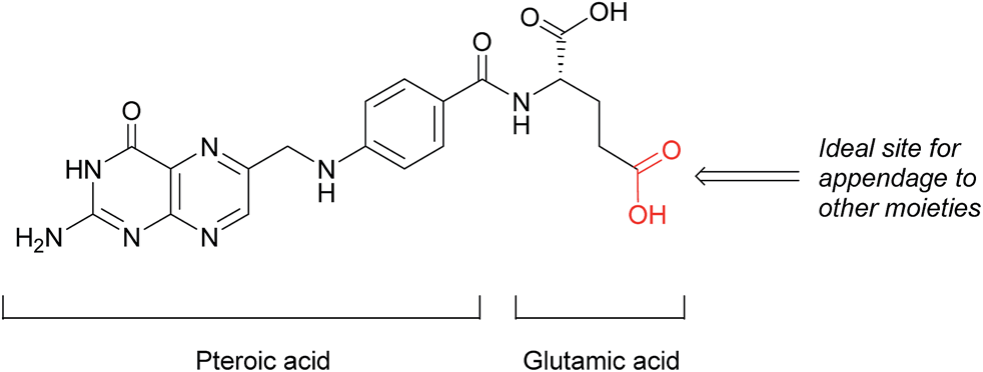
Structure of folic acid (FA). This vitamin's constituent units are pteroic and glutamic acid.

This loss of receptor polarisation, combined with the high binding affinity (*K*_D_ = 0.1–1 nM)^29^ of FRα for oxidised folates, such as folic acid, (FA), has led to the development of drug conjugates with FA as the targeting entity. The benefits of using folic acid in this way are manifold: it is non-immunogenic, cost efficient, has high stability and tissue permeability, possesses a low molecular weight and can be easily conjugated to diverse types of organic molecules, antibodies and nanoparticles.^30^ There are only certain identified positions on the folic acid core scaffold where it can be attached to the rest of the conjugate without compromising the high binding strength to FRα, with one position proving to be the most favoured (Fig. 2).

Folates, once inside the cell, are needed for one carbon methylation reactions as well as the *de novo* synthesis of purines and thymidine, which are in turn required for DNA synthesis and repair. This makes FA integral to the survival of normal cells, but especially important for tumour tissues in order to sustain their rapid, uncontrolled and aggressive proliferation.^10^,^24^,^31^

**Fig. 3:**
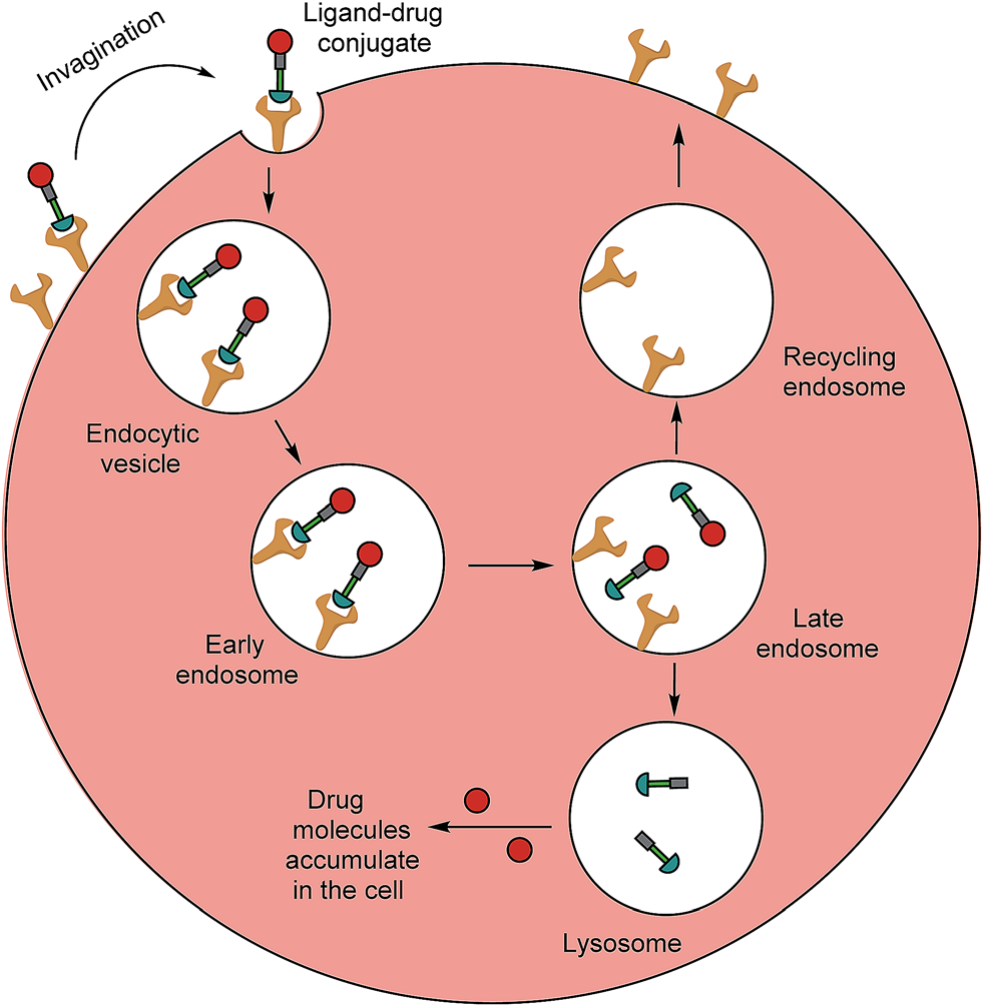
Receptor-mediated endocytosis of a folate–drug conjugate. The construct initially binds to FRα or FRβ, forming an invagination and enclosing the conjugate in the early endosome. A mild drop in pH alters the receptor's conformation, resulting in FA–drug detachment. The late endosome's subsequent fusion with the lysosome leads to degradation of the conjugate and release of the free cytotoxic drug into the cell. The recycling endosome delivers the folate receptors back to the cell surface.^6^

Typically, folates are taken up into cells by FRα and FRβ *via* receptor-mediated endocytosis (RME).^11^ The folate portion of the conjugate acts as the tumour-targeting ligand and will bind strongly to FRα and/or FRβ receptors on a cancer cell, resulting in subsequent internalisation of the folate–drug conjugate. Once the construct is sequestered within the early endosome, resident proton pumps cause a slight decrease in pH, altering the FR's conformation and allowing the conjugate to detach from the receptor.^24^,^32^,^33^ The late endosome then fuses with the lysosome and intracellular thiols, such as glutathione (GluSH) which can degrade the conjugate by cleaving a self-immolative linker, enable release of the free toxic drug. This lethal payload can subsequently diffuse out of the endosome into the cytosol where it induces cell death; meanwhile, the folate receptors are recycled back to the surface of the cell to engage in further rounds of drug internalisation.^24^ The availability of unoccupied receptors on the cell surface is dependent on the recycling rate of the empty receptors from the endosome, and due to its rapid recycling rate (8–12 h),^24^ the FR has the potential to maximise drug capture and internalisation (Fig. 3).

This ability to attach chemical warheads to ligands that seek out FRα-expressing tumours, confers excellent selectivity to the construct while preserving drug potency, and this approach has led to the development of many small molecule–drug conjugates based on folic acid (FA–SMDCs).

## 2 Small molecule–drug conjugates (SMDCs)

### 2.1 Vintafolide

The most successful FA–SMDC is vintafolide, (formerly EC145): a water-soluble conjugate that selectively delivers the drug desacetyl vinblastine monohydrazine (DAVLBH) to tumours that overexpress FRα.^29^ Preclinical studies have shown vintafolide to bind to FRα with high affinity, and therefore has very specific and potent activity against FRα positive tumour xenografts as opposed to the untargeted DAVLBH.

**Fig. 4:**
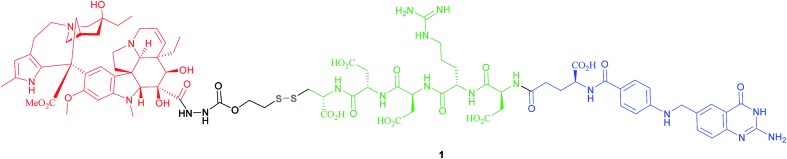
Chemical structure of the folic acid-based SMDC vintafolide **1** is comprised of a folate targeting ligand (blue), a peptide spacer (green), a self-immolative disulfide linker (grey) and the potent cytotoxic drug DAVLBH (red).

The four constituent modules of vintafolide consist of: (1) a folic acid moiety to target FRα, (2) a hydrophilic peptide spacer, (3) a self-immolative disulfide linker, and (4) a microtubule-destabilising drug DAVLBH (Fig. 4).^24^ As folic acid is lipophilic, the spacer serves to ameliorate the overall water solubility of the drug conjugate and in so doing, eliminates non-specific diffusion across cell membranes and ensures cell internalisation *via* RME. Typical examples of spacers commonly employed in FA–SMDCs include polysaccharides, peptides and polyethylene glycol (PEG) chains.^6^,^24^ An additional function provided by the spacer is to physically separate the drug cargo and targeting ligand, thereby minimising steric interference between the two and ensuring the retention of receptor binding affinity for the ligand.^6^,^24^ However, spacer length should not be too great as long, flexible spacers can allow the drug moiety to loop back and interact with the targeting ligand, jeopardising its affinity for the receptor.^7^

Small size (typically lower than 2000 Da) is critical for superior FA–SMDC tumour penetration and rapid systemic clearance.^24^ Possessing a molecular weight of 1917 Da, vintafolide fulfils this criterion and displays a distribution time of 6 min.^14^ This short delivery time indicates rapid uptake of the drug conjugate by FR-positive tumour tissue, which is a desirable characteristic in minimising circulation time, and thus precluding premature drug release. This FA–SMDC is also rapidly cleared from the body (elimination half-life of 26 min) *via* the kidneys and liver.^34^

**Fig. 5:**
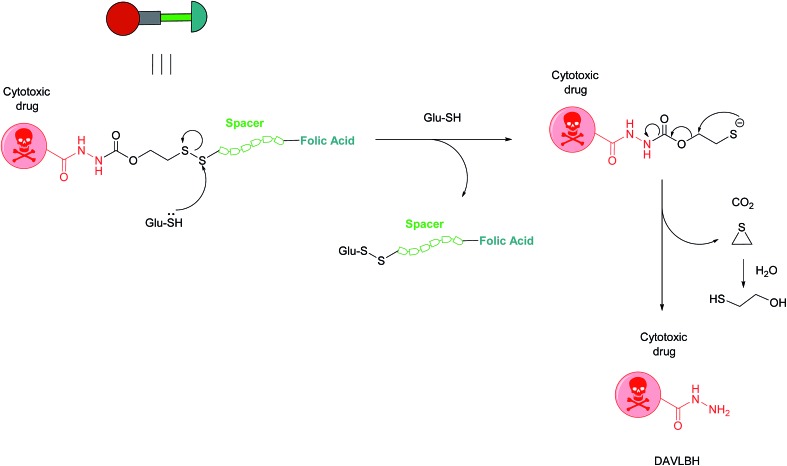
Mechanism of vintafolide drug release in the intracellular milieu. Glutathione cleaves the disulfide linker and the resulting thiolate undergoes a self-immolative, 1,2-elimination reaction to liberate the free drug DAVLBH.^24^

Owing to these attractive and interesting properties, Leamon *et al.* carried out a study aiming to evaluate the impact of altering three out of the four of vintafolide's constituent elements. They demonstrated that varying the spacer composition, provided that it remained hydrophilic, had minimal effect on the potency of the conjugate. In contrast, bioreleasable linkers that can be cleaved by intracellular thiols such glutathione (GluSH) in the endosomal milieu are of critical importance for the conjugate's activity and have by far constituted the most successful approach for triggered drug release within the cell (Fig. 5).^24^ For instance, it was shown that self-immolative disulfide and acyl hydrazone linkers exerted activity both *in vitro* and *in vivo*, whereas vintafolide analogues possessing more stable amide and thioether linkers did not.^29^ Furthermore, it was demonstrated that upon substituting DAVLBH with other clinically approved vinca alkaloid drugs (vincristine, vindesine, vinorelbine and vinflunine) while retaining the cleavable disulfide linker, that vintafolide was the only variant that exhibited biological activity *in vitro* and *in vivo*. This can be rationalised by considering vintafolide's high potency (IC_50_ = 8.44 ± 1.46 nM) as compared to the over ten-fold greater IC_50_ values of all the other drugs (IC_50_ > 100 nM). It was further speculated that the absence of activity observed in the other vinca alkaloid forms could be due to a modification in the chemical structure following disulfide reduction and linker release.^29^

Vintafolide has shown promise, both as a single agent, as well as in combination with doxorubicin in two phase II trials (ovarian and non-small cell lung cancers) and in a randomised open-label phase II study respectively (platinum-resistant ovarian cancer)^29^ and entered phase III clinical trials in 2010 for advanced stage platinum-resistant ovarian cancer.^10^,^35^ However, this FA–SMDC failed to reach the pre-specified criteria for progression free survival and as a result, the trial was prematurely terminated. Despite the expectations to perform otherwise, the phase III trials were unable to solidify the superiority of the targeted FR therapy to conventional chemotherapeutic methods. Future trials and studies must therefore account for an appropriate selection of eligible patients that are likely to benefit sufficiently from anti-FR therapy.

### 2.2 Folate–taxoid conjugate

**Fig. 6:**
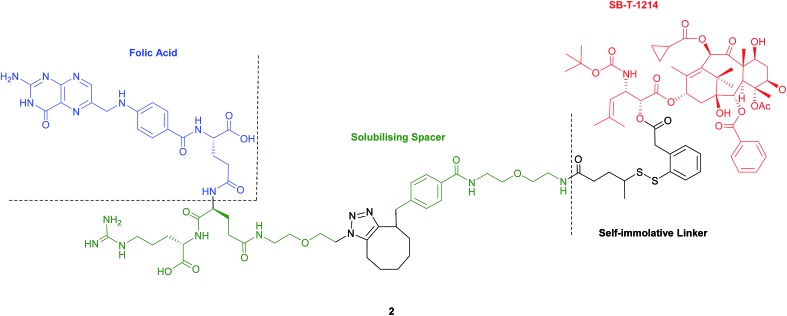
Structure of the folate–taxoid conjugate **2** developed by Seitz *et al.*^1^

Seitz *et al.* have developed a highly potent next-generation folate–taxoid for use against drug-resistant and drug-sensitive cancer cell lines.^1^ This folate–taxoid conjugate incorporates a folic acid targeting moiety and a highly potent taxoid SB-T-1214, which is a derivative of the chemotherapeutic drug Taxol. Similar to vintafolide, this SMDC possesses a self-immolative disulfide linker, and a hydrophilic PEGylated dipeptide spacer (Fig. 6).^1^

*In vitro* analysis was carried out to compare the activity of the taxoid conjugate **2** and free SB-T-1214 in FRα-positive and FRα-negative cells. As expected, free SB-T-1214 was highly potent against all cell lines. Conversely, taxoid conjugate **2** exhibited appreciable cytotoxicity against the FRα-positive cell lines, displaying IC_50_ values more than three times smaller than those observed for the FRα-negative cells. This notable potency has been ascribed to the uptake of the folate–taxoid **2** occurring *via* RME, an internalisation pathway unaffected by the folic acid naturally present in the cell culture medium, which suggests that folic acid required for cell growth is principally shuttled into cells through folate transport proteins *in lieu* of RME. Further, taxoid conjugate **2** also exhibited an over 1000-fold decrease in toxicity against healthy cells compared to the free drug. As with vintafolide, the cytotoxic activity of **2** stems from intracellular GluSH-triggered reduction of the disulfide linker to release the free toxic drug SB-T-1214.^1^ Ideally for maximum biological activity, the drug should be released in its unmodified form, as with conjugate **2**, giving further weight to the aforementioned speculation that the failure of vintafolide analogues may be due to the liberation of a chemically altered payload. Moreover, the efficient release of the chemical warheads is contingent on the GluSH levels present in the intracellular milieu, the concentration of which can vary in different cell lines. It is therefore important to consider this particular variation when selecting tumour cell lines to be targeted by SMDCs whose activity is dependent on the intracellular GluSH concentration. Partly in view of this potential complication/limitation with certain cancer cells and serum stability questionability, FA–SMDCs have been developed where degradation to release free drug is not mediated by intracellular GluSH.

The above examples comprise a small, but representative, selection of FA–SMDCs from a vast field of conjugates that employ a disulfide linker for cytotoxic drug release. It is of particular relevance to highlight that folate conjugates to many other drugs *via* a disulfide linker, such as maytansinoids,^36^ mitomycins,^37^ alkaloid/mitomycins,^38^ tubulysins^39^,^40^ and camptothecins,^41^ have been prepared and appraised.

### 2.3 Dendritic β-galactosidase-responsive folate–monomethylauristatin E conjugate

**Fig. 7:**
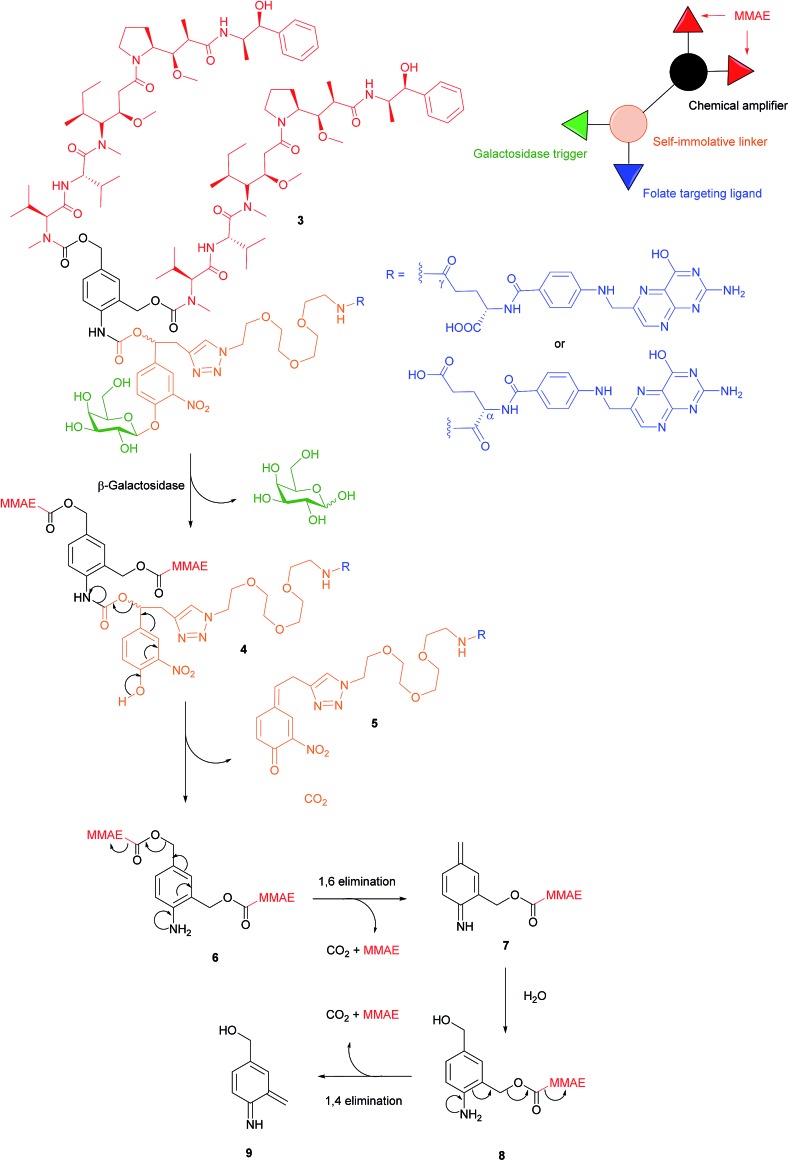
Enzyme-catalysed double drug release mechanism of β-galactosidase-responsive folate–MMAE conjugate **3**.^42^

There are a variety of free thiol-containing compounds present in the blood and as such, the disulfide bond in FA–SMDCs is susceptible to cleavage in circulation by these thiols, potentially giving rise to undesired premature drug release. Consequently, alternative approaches have been developed in which the FA–SMDCs do not possess disulfide linkers, a structural property which would ideally minimise off-target drug liberation in the bloodstream. One such example developed by Alsarraf *et al.* is the β-galactosidase-responsive drug conjugate **3** that delivers the potent antineoplastic drug monomethylauristatin E (MMAE) to cancer cells.^42^ This SMDC consists of a galactoside trigger, phenolic and aniline self-immolative linkers, a folic acid targeting ligand and two MMAE molecules centred around a chemical amplifier, enabling a release of two drug molecules *via* a single internalisation and activation pathway. The warhead release mechanism was studied by incubating folate-conjugate **3** with β-galactosidase at pH 7.2 and at 37 °C. The cleavage mechanism begins with the enzyme-mediated hydrolysis of SMDC **3**'s glycosidic bond, generating a phenol intermediate **4** which undergoes 1,6-elimination and a successive decarboxylation to concomitantly yield quinone **5** and an aniline intermediate **6**. Ensuing 1,6- and 1,4- elimination processes result in the release of two MMAE molecules (Fig. 7).

The breakdown of conjugate **3** was monitored by HPLC and at *t* = 35 min, peaks corresponding to free MMAE, regioisomers of **5** and aniline intermediate **8** were detected. Trace amounts of phenol **4** and dimer **6** were also present in the mixture 35 minutes after the addition of β-galactosidase. Notwithstanding the complexity of the double drug liberation mechanism, MMAE release reached completion at *t* = 2 h, indicating that the process occurs reasonably quickly.^42^ The function of β-galactosidase in this release mechanism, which is found in the cell lysosome, was further confirmed by observing the absence of reaction when the same enzyme was incubated with non-cleavable glucuronide analogues of folate-conjugate **3**.

A 2,3-bis-(2-methoxy-4-nitro-5-sulfophenyl)-2*H*-tetrazolium-5-carboxanilide salt (XTT) cytotoxic assay was then performed using conjugate **3** and its monomeric analogue on FRα-positive cancer cell lines HeLa, SKOV-3 and A2780, but whose FRα expression is significantly lower levels than that of KB cells.^42^ Cell viability of all HeLa, SKOV-3 and A2780 was radically diminished by conjugate **3** (with IC_50_ ranging from 9.62 to 64.51 nM), whereas a *ca.* 2- to 4-fold reduction in cytotoxicity was observed for the same cell lines when incubated with the monomer. This would appear to indicate that the higher levels of MMAE released from the dimer **3** result in increased tumour toxicity. The cytotoxic amplification of the dimer and monomer was then investigated by incubating A2780 cells with equal concentrations of both conjugates. If the β-galactosidase hydrolysed the same amounts of dimer **3** and the monomer, a 2-fold MMAE release would be expected from the former relative to the latter. However, analysis by HPLC/HRMS showed a 4-fold increase of MMAE drug liberation from **3** as compared to the monomer and a proposed hypothesis for this phenomenon is that β-galactosidase released from dead cancer cells can participate in the extracellular activation of non-internalised MMAE conjugates.^42^ This rationale is further supported by studies conducted by Antunes *et al.*, among others, showing that glucuronide prodrugs can be activated by β-glucuronidase liberated from dead tumour tissues.^43^,^44^

A further example of an FA–SMDC that does not bear a disulfide linker and is cleaved by an enzyme is a folate–camptothecin conjugate degraded by the cathepsin B enzyme.^45^ In addition to FA–SMDCs that are cleaved by enzymes already present in the tumour milieu, folate–enzyme conjugates have also been developed to deliver an enzyme to the folate receptor of the tumour cell prior to the administration of a prodrug that is converted to the active form by this enzyme. An example of this therapy utilises penicillin-V amidase and a doxorubicin prodrug.^46^ Consequently, this work paves the way for the development of a new generation of enzyme-responsive FA–SMDCs that could broaden the scope of selectively targeting FRα-expressing tumours. To the best of our knowledge, no FA–SMDCs have been developed to specifically target the FRβ isoform, but as both FRα and FRβ have a similar affinity for folate and internalise *via* RME, there is scope for adapting the technologies applicable to FRα to the FRβ isoform.

### 2.4 Other linker platforms

#### 2.4.1 Boron–nitrogen linker

**Fig. 8:**
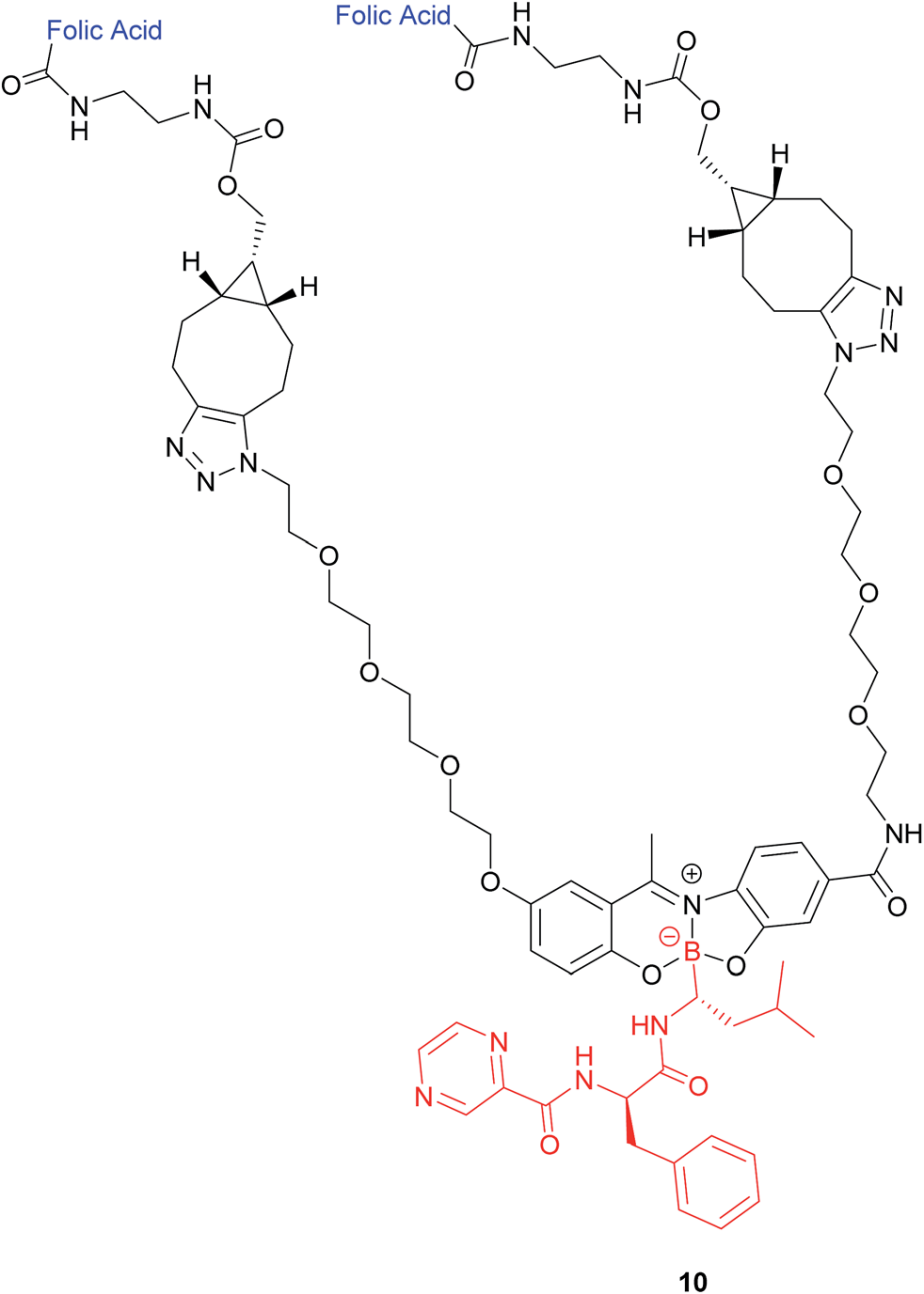
Structure of the boron complex **10** developed by Gois *et al.* consisting of (i) a folic acid targeting moiety (blue), (ii) PEG chains and (iii) the cytotoxic agent bortezomib (red).^47^

In addition to the commonly employed disulfide and carbon-based linkers for drug release inside the cell, the covalent attachment of boronic acids to Schiff base ligands to yield boronate complexes can also be utilised as a platform to selectively deliver cytotoxic drugs to cancer cells. Gois *et al.* designed such a complex (**10**), which comprises the cytotoxic drug bortezomib, PEG chains and folate targeting units (Fig. 8).^47^

**Fig. 9:**
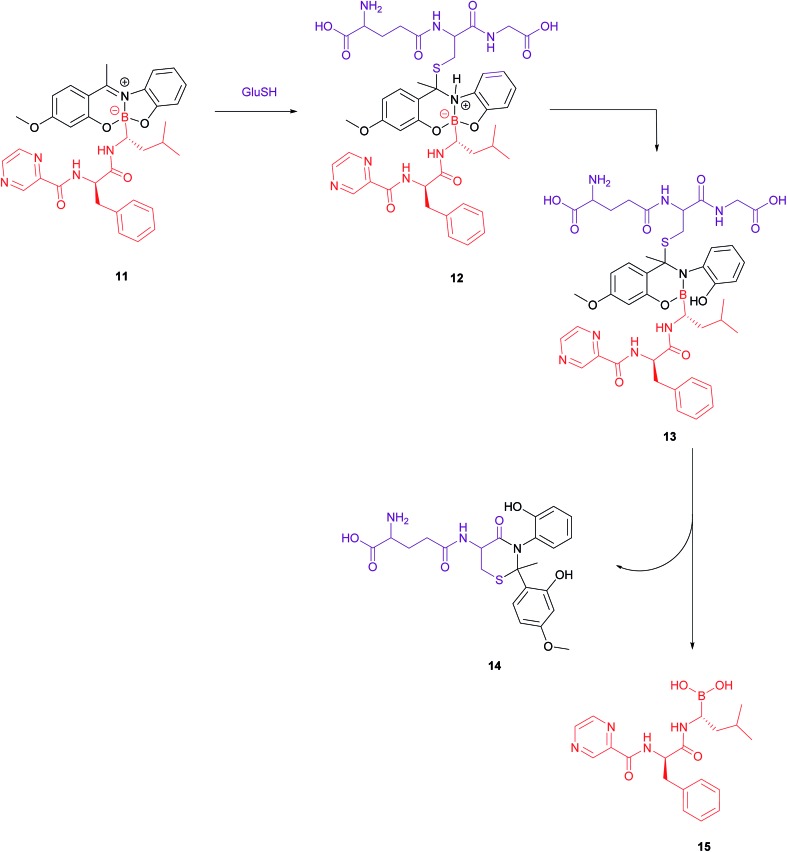
Proposed mechanism for GluSH-mediated release of bortezomib (**15**) from complex **11**.^47^

A bivalent folate targeting moiety was chosen to mimic the bivalent Fab regions present on immunoglobulin Gs (IgGs) that give rise to high affinity and specificity of antibodies for particular antigen epitopes.^47^ Complex **10** exhibited an IC_50_ value of 62 nM against MDA-MB-231 cancer cells, lower than that of free bortezomib, but superior selectivity for these FRα-overexpressing cells as compared to the free drug. As GluSH is present in millimolar concentrations in the cell, Gois *et al.* investigated the GluSH-mediated cleavage mechanism by synthesising complex **11**, a less sterically hindered analogue of complex **10**. The mechanism of drug release, as determined by HPLC, is thought to proceed *via* GluSH addition to the iminium carbon of the complex followed by opening of the five-membered ring and subsequent hydrolysis to promote release of drug **15** (Fig. 9).

#### 2.4.2 Light-triggered drug release

**Fig. 10:**
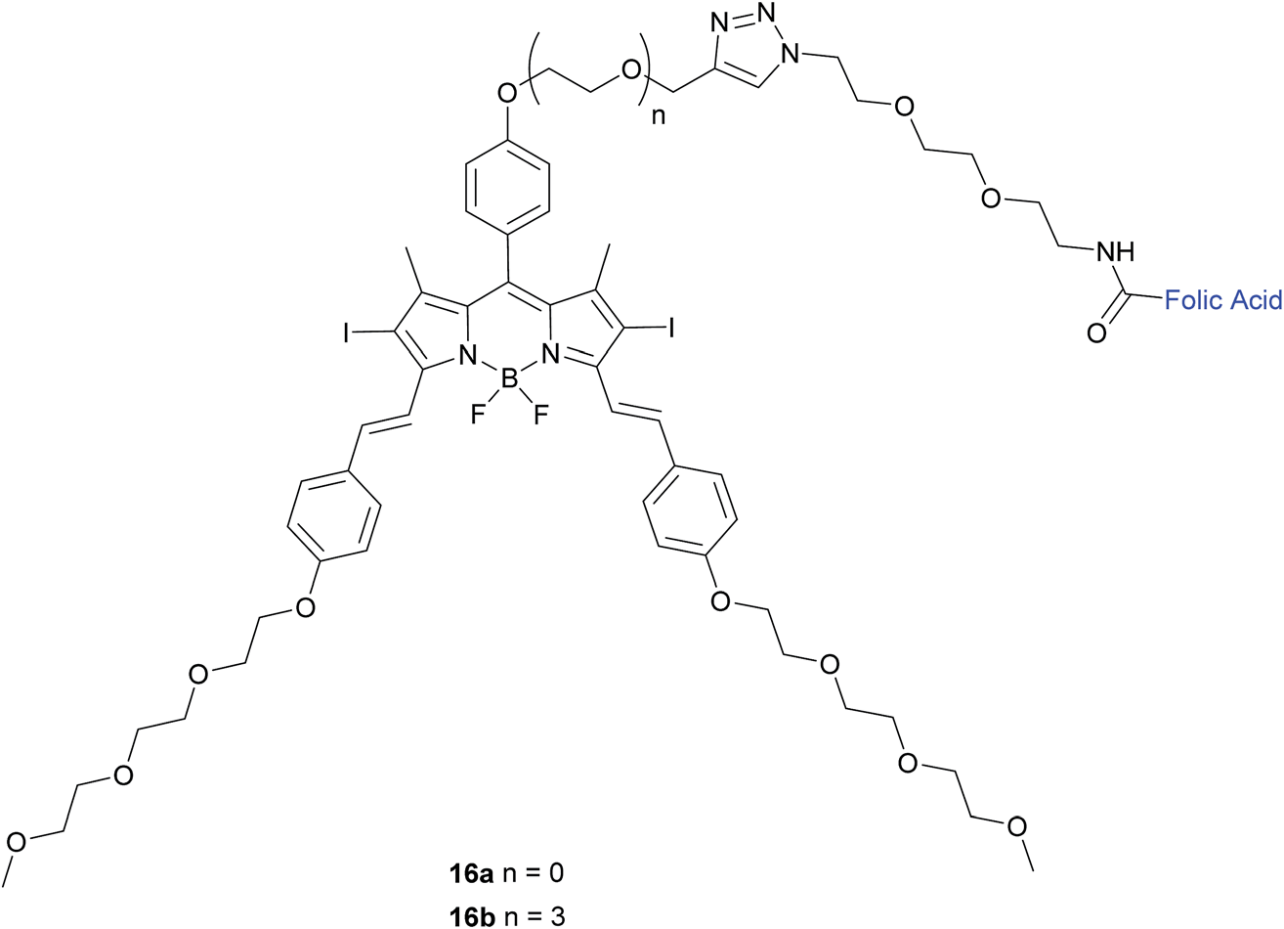
Chemical structure of folate-BODIPY conjugates **16a** and **16b**.^50^

Methods to induce cytotoxicity with light, such as photodynamic therapy (PDT) have also attracted considerable interest for applications in cancer therapy. This technology involves light-mediated activation of a photosensitiser in the presence of oxygen and the subsequent generation of reactive oxygen species that neutralise the cells that have been exposed to the photosensitiser.^48^ Moreover, the advantages of light-based techniques include non-invasive activation and added selectivity from the ease of this medium's spatial and temporal manipulation.^49^ An example of a promising class of photosensitisers are boron dipyrromethene (BODIPY) derivatives that possess attractive optical and photophysical properties as well as displaying high stability in aqueous media.^50^ Ke *et al.* have developed two diiododistyryl folate-conjugated BODIPY-based photosensitisers (**16a** and **16b**) with differing glycol linker lengths (Fig. 10).

The *in vitro* photosensitising ability of **16a** and **16b** was investigated by incubation both with KB human nasopharyngeal carcinoma cells, which have high expression of FRα and with MCF-7 human breast adenocarcinoma cells, which have low expression of FRα.^50^ No cytotoxic activity was detected for either in the absence of light, whereas activity was observed upon the illumination with IR light. Conjugate **16a**, with no triethylene glycol linker, displayed cytotoxic activity 3-fold higher (IC_50_ of 60 nM) than that of **16b** (IC_50_ of 180 nM).

The difference in cytotoxicity can be explained by the observation that **16b** aggregates more in RPMI culture medium than **16a**, probably due to the triethylene glycol linker of the former inducing dipole–dipole interactions in the neighbouring oligoethylene glycol chains.^50^ Thus, conjugate **16a** with the shorter linker is an attractive candidate for use as a photosensitiser against cancer cells in PDT.

Other examples of FA–SMDCs whose cell-killing action is triggered by light include combretastatin A-4 prodrugs activated by visible/near IR,^51^ a folate–doxorubicin conjugate that liberates doxorubicin when irradiated with UV-light^49^ and a folate–chlorin conjugate where the photosensitising ability of the chlorin unit is activated upon irradiation of red light.^48^

As described above, FA–SMDCs represent a varied class of conjugates for targeted drug delivery. Whilst a large number of these platforms have been targeted to FRα overexpression applications, these platforms can readily be applied to FRβ overexpression scenarios (an emerging field) since folic acid binds to both these receptors. SMDCs are not the only group of treatments available for FR positive tumours, and the development of anti-folate antibodies that preferentially target FRα or FRβ with specificity and selectivity (as they do not possess an indiscriminate folic acid targeting moiety) represents an alternative strategy.^10^

## 3 FR-targeted monoclonal antibodies

### 3.1 Farletuzumab (FRα targeted)

Farletuzumab (MORab003) is an example of a fully humanised anti-FRα mAb, weighing 145 kDa and produced in Chinese hamster ovary cells. This antibody has the added advantages of neither preventing the binding of folic acid to the FRα nor blocking FRα-mediated FA-transport into the cell.^10^ The therapeutic potential of farletuzumab has been shown in both *in vitro* and *in vivo* studies. Preclinical *in vitro* studies show that upon binding to FRα on tumour cells, farletuzumab promotes tumour cell lysis by various modes of action, including complement-dependent cytotoxicity (CDC) and antibody-dependent cell-mediated cytotoxicity (ADCC). Other mechanisms by which this anti-FRα mAb suppresses cancer cell proliferation activity include sustained autophagy and disruption of the FRα and lyn kinase interaction which curtails intracellular growth signalling.^10^

Farletuzumab in combination with carboplatin/taxane, followed by single-agent farletuzumab maintenance was employed in a phase II study performed in patients with platinum-sensitive recurrent ovarian cancer.^10^ Single-agent farletuzumab was well-tolerated by the patients, and no additional toxicity was observed when the antibody was administered in combination with chemotherapy. The study also demonstrated an overall improved response rate compared to historical, platinum-based combination chemotherapy regimens.^52^–^55^ Unfortunately, phase III trials in both platinum-resistant and platinum-sensitive ovarian cancer failed to replicate the promising results of the *in vitro* and *in vivo* studies. Results from a large phase III study in patients with platinum-sensitive recurrent ovarian cancer evaluated farletuzumab in combination with carboplatin and taxane, and was compared with carboplatin/taxane alone. Disappointingly, this trial did not meet the primary end point of improving progression-free survival (PFS).^10^,^34^ It is hoped that better patient selection will improve therapy for the future.

### 3.2 IMGN853 (FRα targeted)

In addition to stand-alone therapeutic antibodies such as the aforementioned farletuzumab, antibody–drug conjugates (ADCs), where a cytotoxic agent is covalently linked to an antibody, are now being employed as vehicles for the selective delivery of drugs to tumours. This technology combines the exquisite binding selectivity of antibodies and the potent toxicity of a chemical warhead, whose cell-killing potential is distinct from antibody-dependent cytotoxicity, whilst also minimising off-target toxicity.^56^ This consequently enables the use of drugs that would otherwise be too toxic to be employed in conventional chemotherapeutic regimens. Moreover, the attachment of the cytotoxic agent magnifies the antibody's activity and has the potential to circumvent the rarely curative action of unconjugated antibodies.^57^ As opposed to the short circulation half-life typical of SMDCs, antibodies' large size confers a substantially longer half-life to the ADCs in the bloodstream, which in turn augments the proportion of the administered dose reaching and penetrating the tumour.

**Fig. 11:**
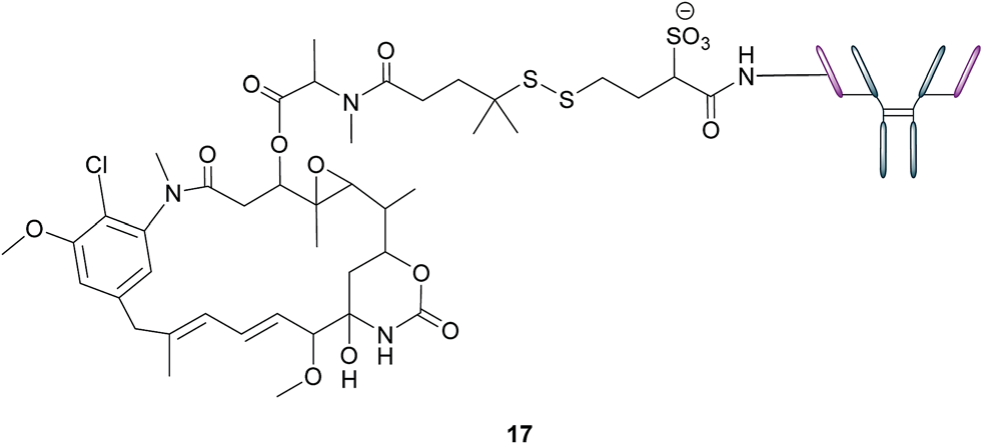
Structure of IMGN853 (**17**). The anti-FRα antibody is conjugated to the DM4 drug *via* a self-immolative disulfide linker.

An example of such a FRα-targeting ADC is IMGN853 (**17**), and it comprises three elements: (1) an anti-FRα antibody that targets the FRα-expressing cancer cells, (2) DM4, an antimitotic agent that inhibits tubulin polymerisation and microtubule assembly and (3) a disulfide-based linker that connects the drug to the antibody (Fig. 11).^34^ As with the FA–SMDCs, IMGN853 binds to FRα, is internalised *via* RME, and ensuing enzymatic degradation of the antibody and linker releases the DM4 drug, which induces cell-cycle arrest and death by disrupting microtubule function. IMGN853 has demonstrated anti-tumour activity^58^,^59^ and is currently being assessed in phase II trials as a single agent and in combination regimens for patients with FRα-positive platinum-resistant ovarian cancer. This ADC represents a first generation construct of its type and there is plenty of scope to refine its chemistry should the clinical trials be unsuccessful.

### 3.3 Antibody m909 (FRβ targeted)

In light of the observation that activated macrophages play an important role in the development and evolution of autoimmune diseases and certain cancers, Feng *et al.* developed an anti-human FRβ-selective IgG1 antibody (m909) and demonstrated its ability to induce lysis in FRβ-expressing cells.^19^

The ability of m909 to bind to cells expressing FRβ was investigated by incubation of the antibody with CHO-hFRβ (FRβ positive) and CHO-K1 (FRβ negative) cell lines. As expected, flow cytometry demonstrated binding of m909 to CHO-hFRβ cells, but none to CHO-K1 cells. This selectivity was further assessed by incubating m909 with KB nasopharyngeal cells, known to express a large amount of FRα on their surface.^60^ No binding of m909 was observed on the KB cells, confirming the selectivity of this antibody for FRβ.

Subsequently, antibody m909's potential to induce ADCC was investigated by incubating m909, as well as a control isotype IgG, with three cell lines which themselves had been pre-incubated with NK cells: CHO-hFRβ (FRβ positive), preB L1.2 (FRβ positive, but lower than CHO-hFRβ) and CHO-K1 (FRβ negative). Gratifyingly, cell lysis induced by m909 was observed at higher levels in CHO-hFRβ than in preB L1.2 and no cytotoxicity was detected in the FRβ negative CHO-K1 cells. The control IgG exerted no lysis in any of the cell lines, even at a concentration of 200 nM.^19^ These results demonstrate that m909 is able to bind to FRβ-expressing cells and mediate ADCC by recruiting NK cells and this antibody has been used to target FRβ positive acute myeloid leukaemia (AML) blasts with chimeric antigen receptor T cells.

### 3.4 Chimeric antigen receptor T cell therapy for AML (FRβ targeted)

Owing to the successful development of anti-FRβ antibody m909, Low *et al.* incorporated it in the development of chimeric antigen receptor (CAR) T cell-therapy to treat AML, as approximately 70% of all AML tumours upregulate FRβ.^11^,^61^,^62^ This treatment consists of attaching the single-chain variable fragment (scFv) of a monoclonal antibody (in this case m909) to T cell receptor signalling domains and in doing so, a patient's own T cells can bind to antigen-positive tumours with antibody-like affinity.^11^

To demonstrate m909 CAR T cells' reactivity, they were incubated overnight with genetically engineered ovarian cancer C30-FRβ cells. Subsequent analysis of the assays showed release of proinflammatory cytokines IFN-γ, IL-2, tumour necrosis factor α and inflammatory protein 1 α.^11^ ELISA analysis was then used to demonstrate the same reactivity by overnight incubation of m909 CAR T cells and control CD19-28Z T cells with FRβ positive AML lines, with the former incubation producing a greater secretion of IFN-γ than the latter. Low *et al.* also upregulated the expression of FRβ on AML cell lines by treatment of all-*trans* retinoic acid (ATRA) and found that IFN-γ secreted by cells pre-treated with ATRA and then incubated with m909 CAR T cells was significantly greater than those without ATRA treatment.^11^

These *in vitro* results were then appraised *in vivo* using THPI (high FRβ expression) AML cells inoculated in mice, with the administration of m909 CAR T cells leading to tumour regression. *In vivo* proliferation of these T cells was demonstrated by treating mice's peripheral blood with human CD3^+^ cells and subsequent administration of m909 CAR T cells. Analysis after 4 weeks of treatment showed significantly higher levels of peripheral blood T cells as compared to the controls, further demonstrating the selective binding of m909 CAR T cells to FRβ.^11^ Thus, this innovative development of anti-FRβ m909 CAR T cells therapy presents a promising platform for the treatment of autoimmune diseases and AML.

Notwithstanding the possible future success of FRα and FRβ-targeting antibodies, the field is still rather narrow. In contrast, the arena of FR-selective nanotechnology is more expansive and versatile.

## 4 Nanotechnology

### 4.1 Nanoemulsions (FRα targeted)

As highlighted above, conventional chemotherapy is limited by a lack of selectivity, and the unwanted side effects caused by the non-specific cellular uptake of platinum-based regimens can be especially problematic. Nonetheless, due to its highly responsive nature, platinum-based therapy is still used as a leading chemotherapeutic agent in almost all stages of ovarian cancer. However, the case for further support of this choice of therapy is waning. For instance, the high frequency of Pt-based treatment cycles often result in acquired drug resistance which can occur *via* the decreased cellular uptake of Pt, which limits the formation of cytotoxic Pt–DNA adducts. Additionally, intracellular GluSH mediates the detoxification of Pt and leads to the inactivation of Pt by the formation of cisplatin–thiol conjugates, thereby preventing cell death occurring after the formation of the lethal Pt–DNA adducts.^63^

**Fig. 12:**
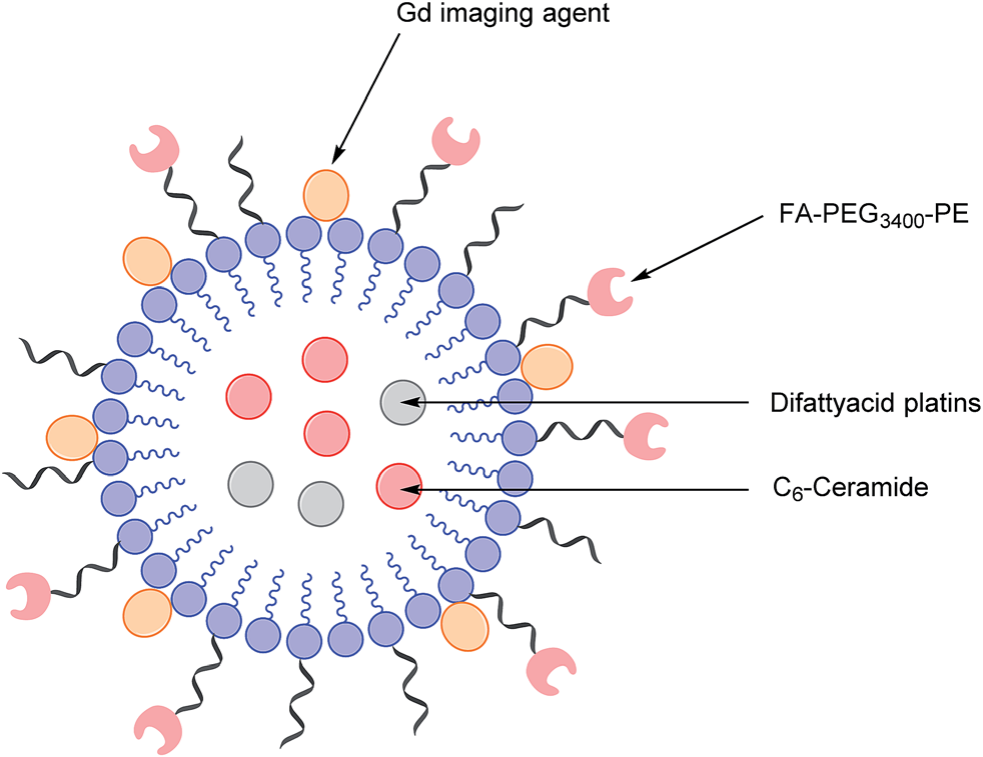
Schematic representation of a NMI-350 nanoemulsion. Difattyacid platins and C_6_-ceramide are encapsulated in the lipid core and lapidated gadolinium and folate are attached to the surface.^21^

In light of this, there is a critical need to modify the Pt therapeutic options currently available. To this effect, Patel *et al.* have reported the synthesis of NMI-350 Pt-theranostic nanoemulsions (NEs). The NMI-350 family is based on naturally occurring polyunsaturated fatty acid (PUFA) rich omega-3 and -6 fatty acid oils and gadolinium (Gd) labelled multicompartmental NEs. Their oily core can encapsulate the cytotoxic and hydrophobic difattyacid platins and C_6_-ceramide, and the NE surface can be employed for the attachment of imaging agents and folate ligands for targeting (Fig. 12).^21^

Through the aforementioned architecture, these NEs allow the controlled delivery of combined chemotherapy and additionally lengthen the blood circulation half-life of Pt to maximise uptake of nanodrug conjugates in malignant cells over a prolonged period of time. Moreover, the synthesis of the difattyacid platinum construct has been greatly improved: Patel *et al.* have developed a synthesis which takes 24 h, as opposed to previously reported procedures requiring 21 days.^64^

**Fig. 13:**
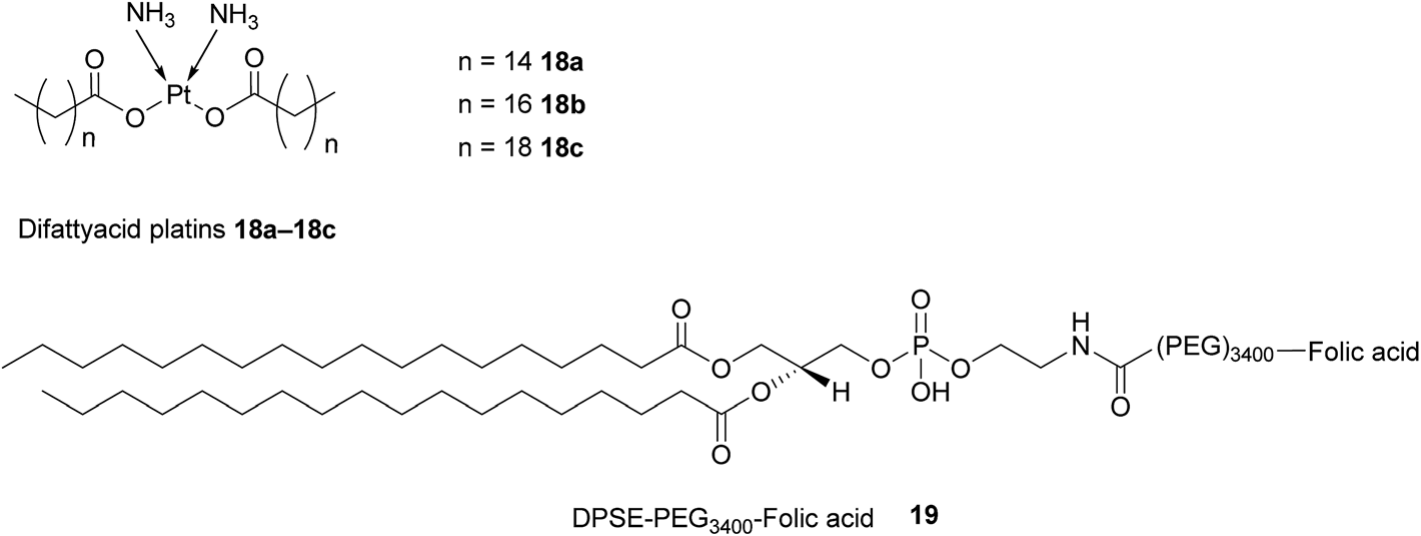
Structure of the difattyacid cisplatins **18a–18c** and the DPSE-PEG_3400_-FA spacer **19**.

Difattyacid platins of different chain lengths were synthesised using this more efficient method and folate was attached to the NE surface *via* a DSPE-PEG_3400_ spacer (Fig. 13). The fully functionalised NEs displayed a particle size in the range 120–150 nm.

FRα-binding efficiency of the NEs was then tested on two FRα-rich cell lines, KB-WT (Pt-sensitive) and KBCR-1000 (Pt-resistant) cell lines and analysed by flow cytometry. Both lines were treated with non-targeted rhodamine labelled NEs (NT-Rh-NE) and FA-targeted rhodamine labelled NEs (FA-Rh-NE), with the latter being functionalised with 100, 300, 1200 and 3600 FA molecules. As expected, cellular uptake in both the lines increased with higher levels of FA conjugation.^21^

The FA-Rh-NE labelled with 300 FA molecules was then selected for a cytotoxic assay due to being the most stable and cost effective relative to the other FA-Rh-NEs. This FA-Rh-NE was compared to cisplatin in a cytotoxic assay using the same Pt-sensitive and Pt-resistant cell lines, and this NE produced a *ca.* 30-fold increase in potency as compared to unconjugated cisplatin. This heightened cytotoxicity has the potential to reverse Pt-resistance and can be ascribed to the synergistic effect of the Pt and the exogenously added C_6_-ceramide. After binding to FRα and ensuing internalisation *via* RME, dissociation of the NE is promoted by the acidic environment of the endosome, permitting the diffusion of the free Pt and C_6_-ceramide across the endosome into the intracellular milieu, where they can exert their cytotoxic activity on chromosomal and mitochondrial DNA. Intracellular depletion of C_6_-ceramide constitutes a resistance mechanism that shifts the equilibrium away from apoptosis in tumour cells.^21^ The addition of the ceramide to NEs serves to combat this resistance mechanism by shifting said equilibrium back towards apoptosis and encapsulation of the ceramide inside the NE shields it from metabolic degradation and inactivation.

The effect of the difattyacid cisplatin aliphatic linker length (C14, C16 and C18) was also evaluated and while the linkers had no effect on the stability of the NEs, the shortest chain **18a** produced the most potent cytotoxic activity. This observation can be rationalised by considering the shortest chain to be the best leaving group during Pt–O bond cleavage, resulting in quicker liberation of reactive Pt which can go then go on to form adducts with the tumour cell's DNA.^21^

### 4.2 Nanotubes (FRα targeted)

Another promising class of nanostructures that is generating increasing interest is that of coordination complex nanoassemblies.^65^ These versatile structures possess desirable characteristics which can be difficult to achieve in other materials. For example, they have the potential to attain various geometries, and influencing tunable binding strength and directionality by choice of ligand.^65^ Wang *et al.* have developed the first example of Ni–folate biomolecule-based coordination complex nanotubes (BMB-CCNTs) of an inner diameter of 5–8 nm and which incorporate FA as a targeting ligand, hydrazine as a linker, Ni as a connector and cisplatin as the cytotoxic agent.^65^ These nanotubes' sufficiently large cavity permits a high drug loading which overcomes the small deliverable payload dose associated with other folate conjugates. Moreover, these nanotubes evade the undesirable accumulation in the kidneys typical of smaller folate–drug conjugates.^65^

**Fig. 14:**
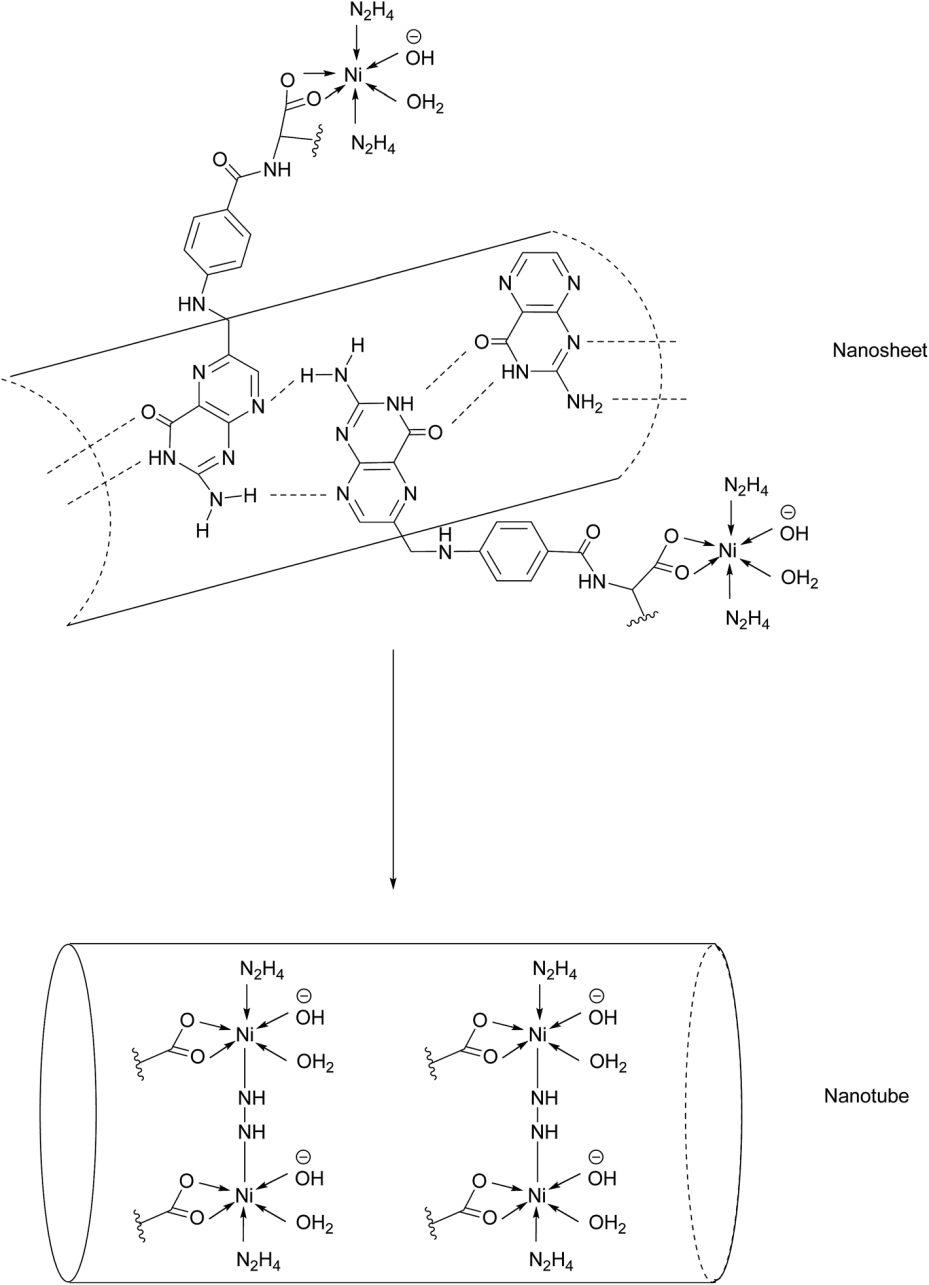
Nanotube formation from nanosheets.^65^

The initial stage of nanotube synthesis comprises the formation of a tape-like structure as the pteroic acid unit of FA can form hydrogen bonds with the pteroic acid moiety of other FA molecules. The glutamic acid portion of FA can then coordinate to Ni^2+^ without compromising the intermolecular hydrogen bonds and hydrazine serves as a bridging ligand between two Ni atoms, resulting in the formation of a nanosheet. The high temperature of this reaction aggravates the relative intermolecular movement of the nanosheets and thus stimulates curling in order to minimise the free surface energy. The high temperatures also promote nanotube formation by the breaking of partial initial bonds and the formation of new ones, with the hydrazine acting as a molecular string, tying the nanosheets into nanotubes (Fig. 14).^65^

A cytotoxic assay was conducted to evaluate and compare the potency of cisplatin-loaded BMB-CCNTs (CDDP-CCNTs), blank BMB-CCNTs, CDDP and free folic acid in three different cell lines: HeLa (high FRα), and low FRα expressing A549 and HELF. As expected the CDDP was toxic to all three cell types. However, despite being less toxic than CDDP, the folate-nanotubes drastically reduced HeLa cell viability far more than HELF and A549. The nanotubes were labelled with fluorescein isothiocyanate (FITC) and their internalisation into HeLa (high FRα) and HELF (low FRα) cells was tracked by confocal laser scanning microscopy. After 24 h of incubation, a strong green fluorescence was observed in the HeLa cells, whereas that of the HELF cells was rather weak, indicating FRα-dependent uptake into the cells. The binding selectivity of the CCNTs to both cell lines was studied by flow cytometry, demonstrating a 34.1% uptake in HELF cells as opposed to 98.6% in HeLa cells. Together with the results of the fluorescence studies, Bio-TEM imaging showed that following uptake into cells *via* RME, the acidic endosomal environment triggers corrosion of the nanotubes and degradation into nanopieces, allowing drug release into the cytosol, giving rise to the inhomogeneous green fluorescence surrounding the nucleus.^65^

To further corroborate the acid-dependent corrosion of the nanotube, the BMB-CCNTs were subjected to endosome-like conditions by soaking in PBS at pH 6.5 for 6 h. As anticipated, the open ends of the nanotubes began to disintegrate into nanopieces. Conversely, the CCNTs' original tubular architecture is preserved when soaked in PBS at pH 7.4, making it an effective targeted delivery system which is stable in blood and only disintegrates inside the cancerous tissue. This original nanotube complex provides insights for creating a novel multi-functional nanomedicine system, which may act as a target seeker and could concomitantly kill multiple malignant cells with a superior efficiency and fewer off-target side effects.^65^

### 4.3 BAL-targeted liposomal doxorubicin delivery (FRβ targeted)

Though the examples of targeted nanotherapy for FRα are more extensive, there are nevertheless nanotechnologies that utilise FRβ as a delivery marker for targeted cytotoxics. One such example developed by Lu *et al.* is an FRβ-targeted liposomal doxorubicin for treating biphenotypic acute leukaemia (BAL).^66^ The group prepared several FRβ-targeted liposomes (f-L-DOX) and varied the mole percentage of FRβ-targeted distearoyl phosphatidylethanolamine (f-PEG-DSPE) in order to assess which lipid analogue constitutes the optimal formulation for therapeutic activity.^66^

Cellular uptake of these liposomes was subsequently studied using fluorometry in order to evaluate whether the lipid mole percentage of f-PEG-DSPE would affect internalisation. As expected, binding and *in vitro* cytotoxicity assays demonstrated that the liposome formulation containing 0.5 mol% f-PEG-DSPE was more efficient in the uptake and more cytotoxic in BAL MV4-11 cells than those of 0.2 mol% and 0 mol%. The optimal mol% was determined to be 0.5 and further augmentation of f-PEG-DSPE (up to 2 mol%) displayed no additional improvement in cellular uptake cytotoxicity.^66^

As previously mentioned, treatment with ATRA can upregulate FRβ expression. In order to evaluate the impact of FRβ upregulation on the cellular uptake and cytotoxicity of these formulations, cells were pre-treated with 1 μM ATRA. The results showed that pre-treating the cells for 5 days with ATRA caused a significant increase in the uptake rate of f-L-DOX by MV4-11 cells. Regarding the cytotoxicity studies, MTT assays showed that f-L-DOX exerted a 4.8-fold increase in cytotoxic activity than L-DOX to MV4-11 cells without pre-treatment with ATRA and 8.6 times greater lytic activity with ATRA pre-treatment. In contrast, the cytotoxic activity of free DOX and L-DOX in MV4-11 cells was not affected by ATRA pre-treatment, suggesting that the impact of ATRA pre-treatment on f-L-DOX-mediated cell death was directly due to FRβ overexpression.^66^ This nanotherapy enables efficient delivery of cytotoxic agents to tumours overexpressing FRβ and could have potential future therapeutic applications in the clinic.

The FRs' upregulation on many different cancer types can be further exploited for diagnostic and imaging purposes.

## 5 Imaging: ^99m^Tc-etarfolatide (FRα targeted)

Appraisal of FRα expression can be a useful diagnostic tool, allowing the FRα status to be monitored throughout the duration of treatment, with several avenues having been explored for FRα detection. However, despite the high specificity and sensitivity of these methods, their clinical use usually requires invasive tissue biopsies, which are typically taken from a single lesion.^67^ Furthermore, the heterogeneous nature of FRα expression on tumours and the changing characteristics of tumours with time makes it difficult to construct an accurate representation of a patient's FRα status, thus generating an incomplete picture. Whole-body imaging that utilises folate radioconjugates can overcome this limitation by providing realtime and non-invasive FRα appraisal for multiple lesions at several time points.^68^,^69^

**Fig. 15:**
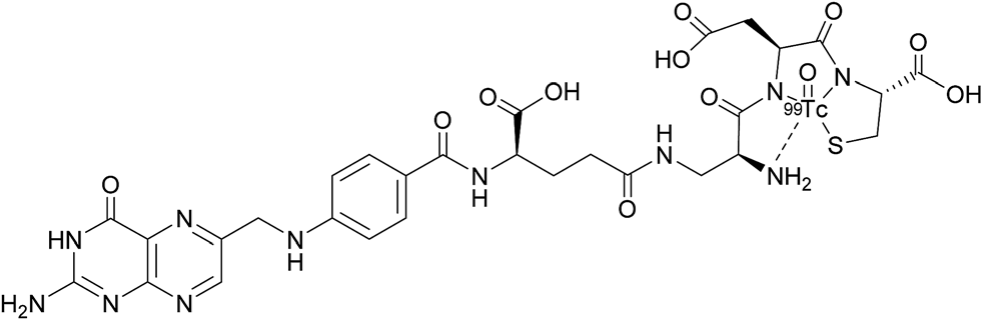
Chemical structure of ^99m^Tc-etarfolatide.

A number of FRα-targeting imaging agents have been evaluated for tumour imaging.^70^–^77^ Etarfolatide (EC20) is one such example and is a folate-targeted radioimaging agent composed of ^99m^technetium (Tc) complexed to folic acid *via* a short non-cleavable peptide linker (Fig. 15).^10^ As opposed to the previously discussed cleavable linkers that are indispensable for drug release in the tumour milieu, EC20's linker is non-degradable as the release of the ^99m^Tc is not a requirement for radiofolate imaging.

^99m^Tc is a frequently employed radiographic tracer, possessing a half-life of 6 h and whose principle form of radioactive decay is gamma emission.^10^ Moreover, ^99m^Tc-etarfolatide displays a strong binding affinity to FRα and tumours that overexpress FRα typically internalise a high proportion of the administered ^99m^Tc-etarfolatide (∼17% ID g^–1^).^70^ Added benefits of this probe conjugate include rapid accumulation at the tumour target site and subsequent swift clearance from the bloodstream *via* the kidneys. This in turn diminishes the non-specific tumour uptake of ^99m^Tc-etarfolatide and permits the quick generation of images.^10^

^99m^Tc-etarfolatide makes use of Tc's optimal single-photon emission computed tomography (SPECT) imaging characteristics, namely, a half-life of 6 h and a photon energy of 140 keV. Consequently, this probe conjugate has been subject to evaluation in numerous clinical trials, including those involving vintafolide, with ^99m^Tc-etarfolatide as a companion imaging agent.^69^,^71^,^78^,^79^ Although no safety concerns have been established in this line of treatment, undesired adverse effects such as lower abdominal pain, nausea and vomiting, have all been identified as being ^99m^Tc-etarfolatide-related, although these were only observed in <1% of patients.^67^

While several phase II trials have demonstrated that ^99m^Tc-etarfolatide imaging can be utilised to determine patients most likely to respond to vintafolide therapy,^68^,^69^ the imaging results and their interpretation can be influenced by physiological factors: principally the observation that ^99m^Tc-etarfolatide is uptaken into the kidneys, bladder, spleen and somewhat into bone marrow. This may interfere with the interpretation of receptor expression in lesions close to these organs and for this reason, small quantities of folic acid are injected prior to ^99m^Tc-etarfolatide administration in order to partially saturate the FRαs.^67^ Another limitation of this probe conjugate stems from activated macrophages (that express FRβ) also internalising ^99m^Tc-etarfolatide, a phenomenon which can result in regions of inflammation or infection falsely appearing as FRα-positive tumour tissue.^67^

Early studies on ^99m^Tc-etarfolatide imaging were constrained by having to employ separate SPECT and computed tomography (CT) imaging, but contemporary SPECT/CT fusion imaging has greatly ameliorated spatial localisation and is able to determine whether tumours are FRα-positive or FRα-negative. ^99m^Tc-etarfolatide has proved to be valuable for the selection of patients likely to respond to treatments targeting the FRα. This probe conjugate has also shown promise for the staging and restaging of tumours, the assessment of disease prognosis and for the identification of patients who could benefit from intraoperative fluorescence FRα imaging to help reveal deep-seated tumours that can evade detection by intraoperative optical imaging due to limited signal penetration in human tissue.^67^^99m^Tc-etarfolatide may also have future applications for the prognosis of FRα-positive ovarian and lung cancer.^80^,^81^

### 5.1 ^68^Ga and ^64^Cu radiofolates (FRα targeted)

**Fig. 16:**
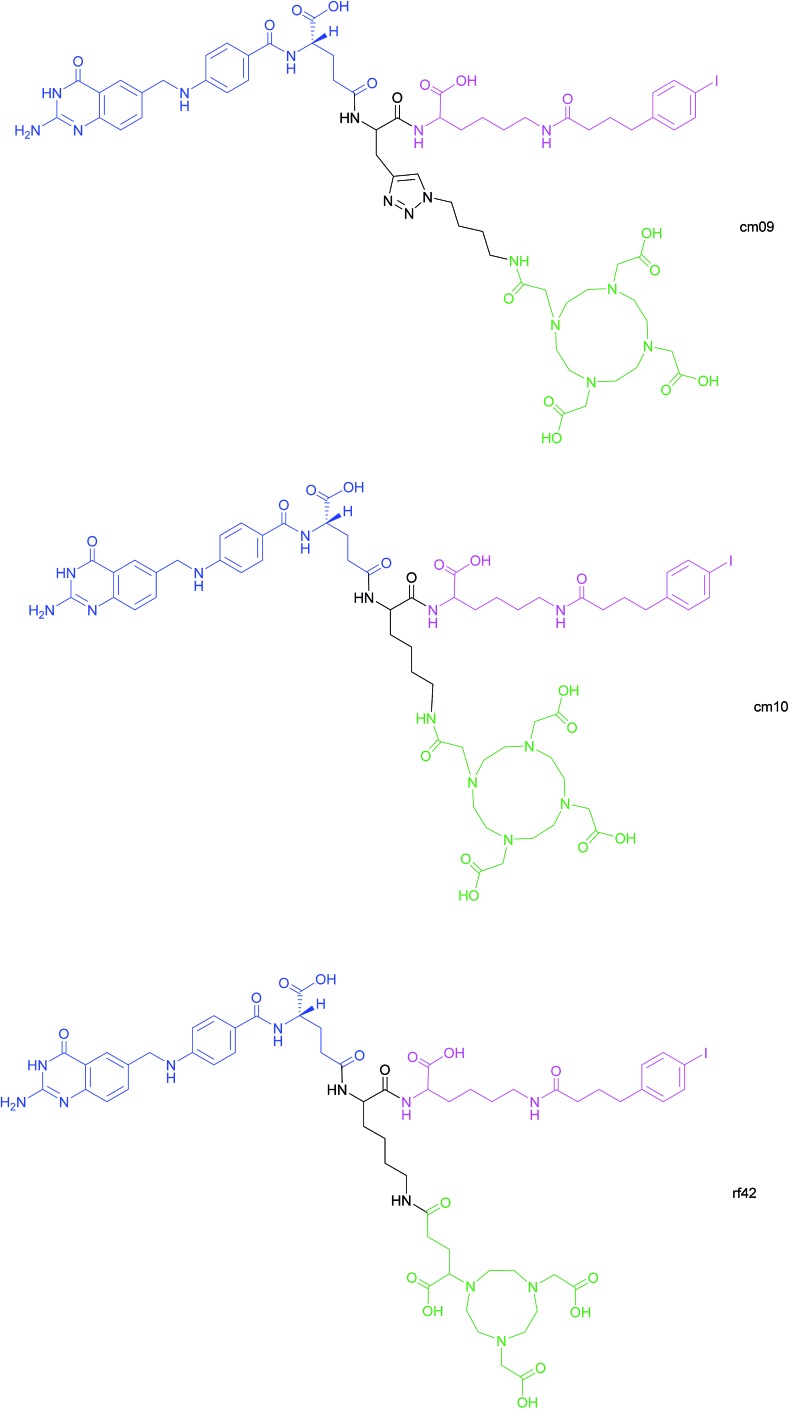
Chemical structures of albumin-binding folate conjugates cm09, cm10 and rf42. They are each composed of three modules: (i) folic acid for FRα-expressing tumour targeting (blue), (ii) an albumin-binding moiety (pink), and (iii) the chelator (green) (DOTA for cm09 and cm10 and NODAGA for rf42) for coordination of ^68^Ga or ^64^Cu. The three functionalities are connected *via* a lysine linker in radiofolates cm10 and rf42, and by a triazole linker in the case of conjugate cm09.^22^,^82^,^85^

Another approach for the imaging of FRα-positive tumours is the use of radiofolates for positron emission tomography (PET). This is a commonly used form of nuclear imaging and in a clinical context, is generally preferred to single-photon emission computed tomography (SPECT) owing to its superior resolution and sensitivity as well as its capability of quantifying the exact levels of accumulated radioactivity.^82^,^83^ Notwithstanding the widespread use of PET imaging, its application to small molecule–folate radioconjugates is limited and stems from their short circulation time in the bloodstream, resulting in high and undesirable renal accumulation of radioactivity. As human serum albumin is the major blood thiol, representing 80–90% of human plasma's thiol concentration,^84^ Müller *et al.* sought to address the problem of renal radioactivity retention by functionalising folate conjugates with an albumin-binding moiety in the hope that this would confer a longer blood circulation time.^85^ It was found that the novel albumin-binding folate radioconjugate (cm09) not only diminished renal radioactivity uptake, but demonstrated the added benefit of greater tumour accumulation.^85^ Folate radioconjugate (cm09) and the next-generation analogue (cm10) have been employed for theranostic and therapeutic applications in preclinical studies, using radionuclides such as ^177^Lu, ^44/47^Sc and ^149/161^Tb (Fig. 16).^86^–^90^

Despite ^68^Ga being the most frequently used radiometal in PET imaging, it possesses a half-life of only 68 min, rendering it non-ideal for chelation to the aforementioned albumin-binding radiofolates. In contrast, ^64^Cu has a half-life of 12.7 h, permitting PET imaging at >24 h post-injection (p.i.) of the folate radioconjugate. Moreover, the positron energy of ^64^Cu is almost as desirably low as that of ^18^F (Eβ–av = 250 keV), which is currently the most commonly utilised clinical PET nuclide.^82^ As the established radiofolates cm09 and cm10 with a 1,4,7,10-tetraazacyclododecane-1,4,7,10-tetraacetic acid (DOTA) chelators, proved to be suboptimal for the coordination of ^68^Ga and ^64^Cu, Müller *et al.* synthesised a novel albumin-binding folate conjugate (rf42) with a 1,4,7-triazacyclononane, 1-glutaric acid-4,7-acetic acid (NODAGA)-chelator, permitting stable chelation to the radiometals ^68^Ga and ^64^Cu (Fig. 16).^82^

The coordination of the radionuclides to rf42 proved to be very efficient, with both ^68^Ga-rf42 and ^64^Cu-rf42 displaying a radiochemical purity of >95% after 10 min at room temperature. To assess the protein binding of these conjugates, the prepared ^68^Ga-rf42 and ^64^Cu-rf42 were then incubated with human plasma and the filtered fraction of the plasma samples was found to contain negligible levels of radioactivity, suggesting that the majority of both radiofolates was bound to plasma proteins, with no significant difference in bound fraction between ^68^Ga-rf42 (98.0 ± 0.2%) and ^64^Cu-rf42 (96.3 ± 1.3%).^82^ A control experiment, consisting of incubation of ^68^Ga-rf42 and ^64^Cu-rf42 in PBS (pH = 7.4), found that the subsequent filtrate contained >95% of the loaded radioactivity. This confirmed that the low detected levels of radioactivity in the plasma samples incubated with ^68^Ga-rf42 and ^64^Cu-rf42 are due to the binding of these radiofolates to plasma proteins. Moreover, the protein-bound fraction of both ^68^Ga-rf42 and ^64^Cu-rf42 was observed to be higher than that of ^68^Ga-cm10 and ^64^Cu-cm10.^82^

Cellular internalisation of ^68^Ga-rf42 and ^64^Cu-rf42 was performed in KB cells using a γ-counter, and shown to be high, with an internalised fraction of 30% and 55% respectively relative to the totally bound ^68^Ga-rf42 and ^64^Cu-rf42. Samples coincubated with excess folic acid demonstrated a dramatic reduction in radioactivity (<0.2% of total added radioactivity), indicating that the radiofolates are internalised *via* RME.^82^*In vivo* biodistribution studies in mice showed rapid tumour uptake of ^64^Cu-rf42 after 4 h (14.52 ± 0.99% IA g^–1^) and 50% of the maximum radioactivity was still present in the tumours 72 h p.i. of the radiofolate. Elevated tumour uptake was also observed for ^68^Ga-rf42 after 4 h (11.92 ± 1.68% IA g^–1^), though it was lower than that of ^64^Cu-rf42. Off-target radioactivity was detected in the kidneys and salivary glands. The albumin-binding moiety of the conjugates also gave rise to relatively high initial blood radioactivity which cleared comparatively slowly.

PET/CT imaging was then performed on mice bearing KB tumours at 2 h p.i. of ^64^Cu-rf42 and ^64^Cu-cm10. Both radiofolates showed radioactivity in tumours and the kidneys, but the tumour uptake of ^64^Cu-cm10 was lower than that of ^64^Cu-rf42. No difference in tumour uptake was observed for ^68^Ga-rf42 and ^68^Ga-cm10. PET/CT imaging also showed that mice coinjected with excess folic acid displayed marked reduction in tumour radioactivity.^82^ Furthermore, ^64^Cu-rf42's longer half-life allowed PET/CT imaging to be performed on mice up to 72 h p.i. and found that although maximum tumour uptake at 24 h p.i. was similar for ^64^Cu-rf42 and previously reported ^177^Lu-cm09, blood retention of the former was twofold higher at 24 h p.i. and more than four-fold higher at 72 h p.i. than the latter. These results indicate the superiority of the NODAGA chelator relative to the DOTA, and the promising features of ^64^Cu-rf42 that allow PET/CT imaging at longer time points than is possible with ^68^Ga-rf42.^82^

### 5.2 Fluorescent off–on nanoprobe (FRα targeted)

In addition to the previously mentioned PET, CT and SPECT, fluorescence imaging based on nanomaterials has recently emerged as an attractive prospect owing to the relative ease with which it is possible to multi-functionalise a nanoprobe. Fluorescence spectroscopy itself is an imaging technique that possesses exceptional qualities in regards to molecular and cellular imaging, namely, high spatiotemporal resolution and sensitivity.^91^–^93^ However, as the majority of nanomaterial-based fluorescent systems are in a permanently-on state, they are commonly afflicted with drawbacks such as a low signal/background ratio and false positives stemming from non-specific uptake of the nanoprobe on the surface of non-target cells.^94^

**Fig. 17:**
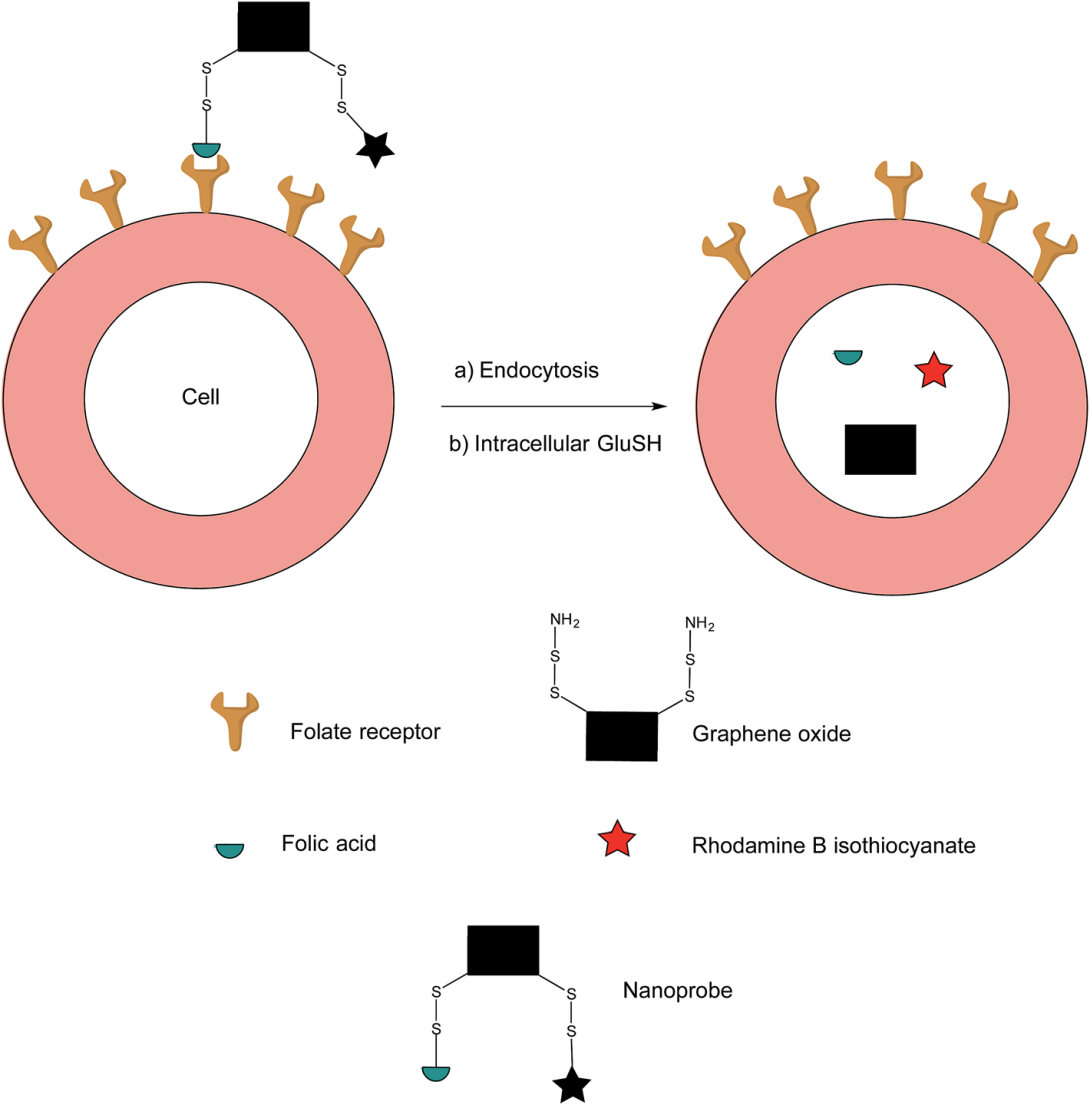
Fluorescence off-on response of nanoprobe in FRα-positive cell.^91^

In an effort to address these issues, Feng *et al.* have developed an off–on nanoprobe, which only fluoresces once bound and internalised in the target cell.^91^ The structure of this nanoprobe consists of three modules: (i) rhodamine B (RB), a fluorochrome that minimises false positive signals by virtue of being impervious to biological environments, (ii) folic acid (FA) as a cell-targeting moiety and (iii) graphene oxide (GO) which is both an efficient quencher for organic fluorochromes and biologically compatible. The GO surface was coated with amino disulfide bonds, with the amino portion of the linker allowing conjugation to FA and RB (Fig. 17).^95^–^100^

Ideally, the intact nanoprobe should display negligible to weak fluorescence due to the quenching action of the GO. Once the probe is internalised by the cell, intracellular GluSH should cleave the disulfide bond, thereby liberating the RB from the GO and generating fluorescence.^91^

The sensitivity of the nanoprobe was investigated by incubation in PBS (pH = 7.4) at 37 °C with two different concentrations of GluSH, 1 mM and 10 μM, the lowest intracellular and the highest extracellular concentration respectively. Incubation with 1 mM GluSH displayed a large increase in fluorescence, suggesting efficient cleavage of the nanoprobe's disulfide bonds by GluSH. On the contrary, incubation with 10 μM GluSH produced a rather weak fluorescent response with a low signal/background ratio. This significant discrepancy in fluorescence is an attractive feature for sensitive intracellular imaging.^91^ The cleavage action of thiols was confirmed through separate incubations of the nanoprobe with 1 mM cysteine and homocysteine, both experiments showing increases in fluorescence. The essential incorporation of the disulfide bond for fluorescence was also investigated by observing a very small increase in fluorescence when incubating GluSH with a control nanoprobe containing an alkane linker *in lieu* of a disulfide connector.

The hypothesis that this nanoprobe is internalised *via* RME was confirmed by incubating the nanoconstruct with FRα-overexpressing HeLa cells. Confocal laser scanning microscopy showed a steady increase of fluorescence which levels out after 3 h, suggesting that total degradation of the nanoprobe is completed after this time. Separate experiments where HeLa cells were pre-treated with (i) excess FA and with (ii) the nanoprobe lacking the FA moiety, showed weak fluorescence after subsequent incubation with the unmodified nanoprobe, corroborating internalisation *via* RME. This internalisation pathway and thus the selectivity of the nanoprobe were further validated by incubating the nanoprobe with HeLa (FRα-positive) and NIH-3T3 and MCF-7 (FRα-negative) cell lines. As expected, only HeLa displayed fluorescence ∼16 and 7 times higher relative to NIH-3T3 and MCF-7 cells respectively.^91^

An additional challenge in the cancer diagnosis field is the distinction of tumours that are morphologically similar. As HeLa and MCF-7 cells are akin in their morphologies, both lines were co-incubated for 12 h with the HeLa cells being subjected to an initial 30 min staining with a fluorescent dye in order to efficiently identify both cell types. Once the co-incubation was complete, the nanoprobe was added to the cell mixture and incubated for 3 h. Confocal laser scanning microscopy revealed a strong fluorescent signal in HeLa cells, whereas that detected from the MCF-7 cells was negligible, confirming this nanoprobe's ability to differentiated between FRα-positive and FRα-negative cells lines as well as those with similar morphologies, properties which are promising for cancer diagnostics.^91^ Future work on this nanoprobe could include incubation with human plasma to appraise the nature of its stability in blood-mimicking conditions.

The majority of these imaging technologies have been targeted/focused on FRα-expressing tumours. Although FRβ imaging has been studied in the context of many inflammatory diseases,^13^,^19^,^101^–^104^ there does not appear to be as wide a range of imaging techniques specifically geared towards FRβ-expressing cancers. Sun *et al.* have investigated the use of folate–FITC as a fluorescent imaging agent for the visualisation of FRβ-expressing TAMs in head and neck squamous cell carcinoma.^17^ However, this technique appears to possess limited clinical value: TAMs penetrate tumours in a non-uniform manner and therefore targeting of folate–FITC to cells in the tumour microenvironment leads to heterogeneous fluorescence. Although not detrimental for the detection of the tumour, this heterogeneity has limitations in the intraoperative imaging of the tumours. Additionally, due to the poor tissue penetration (a few millimetres)^105^ of folate–FITC, applications for the use of this fluorophore are limited, but could be addressed by those that emit in the near infrared (NIR) region instead.

## 6 Conclusions

A variety of folate receptor targeting constructs have been developed against FRα-expressing tumours, each with their own advantages and limitations (Table 1). SMDCs, where the cytotoxic drug is linked to a folic acid tumour-targeting moiety, have perhaps attracted the most interest. Within this area, a number of technologies have been explored for drug release, including boron-thiol based, light-triggered and enzyme-cleavable strategies, as well as the classical disulfide linker model. In particular, the disulfide linker-bearing SMDC vintafolide has been investigated in clinical trials, making it as far as phase III. These strategies and platforms are also amenable to FRβ-expressing tumours (as folic acid binds both FRα and FRβ efficiently) and represent an exciting emerging field. Complementary and orthogonal targeting constructs such as the FRα-specific mAb farletuzumab have also been investigated. Whilst this mAb experienced limited success as a single agent, the drug-conjugated antibody (IMGN853) is showing far more promise and is currently being investigated in ongoing phase II studies. Nanoparticle-based constructs such as nanoemulsions and nanotubes are also able to exert cytotoxic activity as well as enabling higher drug loadings than SMDCs. In addition to cell death mediated by therapy-based FRα-targeted modules, the overexpression of FRα has also been exploited as a biomarker in fluorescence, PET and SPECT imaging; typically employing similar constructs to therapy approaches but with a non-cleavable linker. The folate-conjugated imaging agent that has progressed the furthest, ^99m^Tc-etarfolatide, has been utilised as a companion agent in vintafolide's clinical trials and provides an accurate assessment of response to FRα-targeted therapies. These folic acid-based targeting methodologies can also be applied to FRβ-expressing tumours. In the context of antibody-based technologies, the m909 CAR T cell therapy has led to *in vivo* AML tumour regression, while in the field of nanotherapeutics, the FRβ-targeted liposome f-L-DOX has demonstrated the ability to induce cytotoxicity in FRβ-positive BAL cancer cells. In terms of imaging, folate–FITC has been used to optically visualise cancers, but it is associated with various disadvantages, with NIR technology having the potential to supersede it in the future.

**Table 1:** A table summarising the different types of FRα- and FRβ-targeting platforms with focus on structure, applications, and their various advantages and limitations

Platforms		Structure	Applications	Advantages	Limitations
Small molecule conjugates (SMCs)	Imaging (applicable to FRα and FRβ expressing tumours)	Imaging agent linked to FA *via* non-cleavable linker or chelation	Disease diagnosis, prognosis, monitoring	Facile tumour penetration due to small size, convenient chemical synthesis, short circulation time, rapid systemic clearance	Undesirable accumulation of drug (SMDCs) and often radioactivity (imaging) in liver and kidneys, small deliverable dose
	SMDCs (only exists for FRα expressing tumours currently)	FA linked to a cytotoxic drug *via* cleavable linker	Tumour-targeted therapy		
Antibody-based	mAbs (tuneable to FRα and FRβ expressing tumours)	Engineered anti-FRα and anti-FRβ antibodies		Exquisite selectivity, used alone or in combination therapy, vehicles for drug delivery to tumour site	Expensive, potentially immunogenic, poor tumour penetration due to large size, long systemic circulation time of ADC may lead to potential off-site toxicity
	ADCs (only exists for FRα targeting currently)	Anti-FRα antibody linked to a cytotoxic drug *via* a cleavable linker		Allows the use of very potent cytotoxic drugs, minimal off-target toxicity	
Nano-based (tuneable to FRα and FRβ expressing tumours as folic acid is used for targeting)		Cytotoxic drug encapsulated within the nanoparticle core, external FA targeting moiety		High drug loading capacity, minimal off-target toxicity (in theory), encapsulation allows use of very potent cytotoxic drugs, biocompatible	Convoluted synthesis, difficult purification and storage

In summary, at present there exists a plethora of linker technologies available for efficient drug release and tumour-imaging for disease diagnosis, prognosis and monitoring in the field of FR targeting. Albeit with some exceptions along the way, considerable recent progress has been made. With all the lessons learned from both the successes and failures, and with several new sophisticated linkers at the fore, there is great promise in how the next-generation of FR-targeted constructs is shaping up.

## Conflicts of interest

There are no conflicts to declare.
